# Clinical and psychosocial characteristics of patients attending a suicide prevention outpatient clinic in Italy: a real-world descriptive study

**DOI:** 10.1192/j.eurpsy.2026.11984

**Published:** 2026-07-31

**Authors:** S. Sarubbi, M. Penzo, P. de Roberto, G. Di Biase, I. Porta, P. Zambrano, M. Migliorati, E. Rogante, D. Erbuto, M. Pompili

**Affiliations:** 1 Department of Neurosciences, Mental Health and Sensory Organs, Faculty of Medicine and Psychology, Suicide Prevention Centre, Sant ‘Andrea Hospital, Sapienza University of Rome; 2 Psychiatry Residency Training Program, Faculty of Medicine and Psychology, Sapienza University of Rome, Psychiatry Unit, Sant’Andrea Hospital, Rome, Italy

## Abstract

**Introduction:**

Suicide is a major global public health concern with multifactorial origins. Accurate assessment of suicide risk and its determinants, together with standardized clinical practices, is essential for effective prevention. Despite the progressive understanding of its dynamics, suicide still causes hundreds of thousands of deaths each year. Our specialized outpatient service implements an evidence-based, intensive follow-up model aimed at delivering individualized, multimodal care to reduce the overall incidence of suicide and suicide attempts. Real-world descriptive studies play a crucial role to define risk profiles and guide the aim of defining personalized prevention strategies.

**Objectives:**

This study aimed to describe the sociodemographic, clinical, and life-history characteristics of patients assessed at a suicide prevention outpatient clinic, outlining a comprehensive profile of individuals at risk and identifying potential intervention targets.

**Methods:**

Data were collected from 105 patients consecutively assessed at a specialized suicide prevention clinic. Sociodemographic, clinical, and psychosocial variables were obtained through structured interviews and medical records, including psychiatric and medical comorbidities, substance use, adverse childhood experiences, suicidal and non-suicidal self-injurious behaviors, and recent life stressors. Descriptive analyses were performed using IBM SPSS Statistics v.30.

**Results:**

The sample (53.3% female) showed that most patients lived with a partner (44.1%) or alone (28.4%) and were employed (52.4%). Nearly half had completed upper secondary education (46.7%), and 27.6% held a university degree. Family psychiatric history was reported by 58.3%, mainly mood (35.4%) and anxiety (7.1%) disorders. Medical comorbidities were present in 70.6%, particularly endocrine (19.6%) and neurological (17.6%) conditions. Substance use was reported by 21.6%, alcohol by 32.7%, and tobacco by 46.1%. Adverse childhood experiences were found in 28.0%, mainly neglect (17.0%), physical (6.0%), and sexual abuse (5.0%). At evaluation, 43.3% had previous suicide attempts, and 46.6% had prior psychiatric hospitalizations. Non-suicidal self-injury occurred in 17.2%. Major stressors included relational (28.4%), economic (23.5%), and bereavement-related (36.9%) factors.

**Conclusions:**

Results highlight a clinical population characterized by high rates of psychiatric and medical comorbidities, prior suicide attempts, biographical vulnerabilities and psychosocial adversity. These findings underscore the complexity of patients at suicide risk and the need for comprehensive, tailored, and multidisciplinary intervention strategies.

**Disclosure of Interest:**

None Declared

